# In silico assessment of the dosimetric quality of a novel, automated radiation treatment planning strategy for linac-based radiosurgery of multiple brain metastases and a comparison with robotic methods

**DOI:** 10.1186/s13014-018-0997-y

**Published:** 2018-03-15

**Authors:** Krzysztof Slosarek, Barbara Bekman, Jacek Wendykier, Aleksandra Grządziel, Antonella Fogliata, Luca Cozzi

**Affiliations:** 1 Department of Radiotherapy Planning, Maria Sklodowska Curie Memorial Cancer Center and Institute of Oncology, Gliwice, Poland; 2 Department of Medical Physics, University of Silesia, Katowice, Poland; 3 Radiotherapy and Radiosurgery Department, Humanitas Clinical and Research Hospital, Rozzano, Italy; 4 Department of Biomedical Sciences, Humanitas University, Rozzano, Italy

**Keywords:** HyperArc, CyberKnife, VMAT, Multiple brain metastases, Radiosurgery

## Abstract

**Background:**

To appraise the dosimetric features and the quality of the treatment plan for radiosurgery of multiple brain metastases optimized with a novel automated engine and to compare with plans optimized for robotic-based delivery.

**Methods:**

A set of 15 patients with multiple brain metastases was selected for this in silico study. The technique under investigation is the recently introduced HyperArc. For all patients, three treatment plans were computed and compared: i: a HyperArc; ii: a standard VMAT; iii) a CyberKnife. Dosimetric features were computed for the clinical target volumes as well as for the healthy brain tissue and the organs at risk.

**Results:**

The data showed that the best dose homogeneity was achieved with the VMAT technique. HyperArc allowed to minimize the volume of brain receiving 4Gy (as well as for the mean dose and the volume receiving 12Gy, although not statistically significant). The smallest dose on 1 cm^3^ volume for all organs at risk is for CK techniques, and the biggest for VMAT (*p* < 0.05). The Radiation Planning Index coefficient indicates that, there are no significant differences among the techniques investigated, suggesting an equivalence among these.

**Conclusion:**

At treatment planning level, the study demonstrates that the use of HyperArc technique can significantly improve the sparing of the healthy brain while maintaining a full coverage of the target volumes.

## Background

The high incidence of (multiple) brain metastases is a heavy burden to any clinical radiation oncology department and requires the development of appropriate and tailored strategies for their treatment. If whole brain irradiation is consolidated and generally accepted as well as the use of stereotactic radiosurgery (SRS) for solitary or few (up to three) lesions are consolidated practice, the use of SRS to treat multiple lesions took longer to reach a sufficient level of evidence. Nevertheless, its considered quite acceptable in the modern management of patients with brain metastases [1–5] although care should be given to the selection of the techniques and of the patients.

Due to the anatomical complexity of the brain, several trade-offs between target coverage and OAR sparing might arise. The use of a coplanar approach to the arc geometry leaves space for improvement. Some groups explored the possibility to deliver modulated treatments (with fixed beams or arcs) with conventional c-arm linear accelerators using most of the 4π space, i.e. making extensive use of non-coplanar beam arrangements and creating complex delivery trajectories for the couch-gantry-collimator system around the patient [6–12]. These investigators focused on stereotactic irradiation in the brain, lungs and prostate and have shown that significantly sharper dose gradients can be achieved with this approach. The original investigations published provided evidence of benefit and proof of principle for smaller tumors.

The aim of this study was to explore a practical implementation of a kind-of 4π technique in a clinically released treatment planning system, the HyperArc (HA). A new dedicated optimization engine and an automated planning procedure was recently released for clinical use. This approach is based on the seminar work of the group of the University of Alabama [13, 14] and combines the use of multiple non-coplanar Volumetric Modulated Arc Therapy (VMAT) with a single isocenter to some dedicated optimization strategies. We aimed to appraise and report about the quality of the treatment plans achieved with this technique (mono-isocentric by definition) and to compare this with respect to plans optimized for a robotic delivery with Cyberkinfe (CK), “multi-isocentric” by definition. In fact, if some extensive literature exists in the discussion of CK, even in comparison with VMAT (in its RapidArc (RA) form) [15–23], this might result among the first reports comparing HA and CK for multiple brain metastases. Non-coplanar RA for multiple brain metastases, was appraised also by Liu [24] in comparison with Gamma Knife based plans demonstrating an improved level of conformity and a reduced involvement of healthy brain. As an extra comparison and a kind of internal benchmark, we added in the study also plans optimized with conventional VMAT approach, obtained without the special planning tools available for the HA plans.

## Methods

### Patients and treatment plans

A group of 15 patients, previously treated for multiple brain metastases, were selected for this retrospective, in silico study. The number of lesions ranged from 3 to 8 (3 lesions: 11 patients, 5,6 or 8 lesions: one patient each) For each case, the treatment plans were optimized and computed with the dose prescription chosen for the clinical treatment which ranged from 10.0 to 24.0 Gy depending on the lesion location and the presence of nearby dose-limiting organs at risk. The clinical delivery (with CK) occurred in 1 fraction for 7 patients, in 2 fractions for 6 patients and in 3 fractions for 2 patients. For the planning study, a single fraction was considered in all cases. As usual in a planning comparison study, to avoid any bias derived from different normalization or prescription methods among techniques, the same strategy was applied to all cases for all techniques. The planning strategy required full coverage with a dose normalization set so that: V_100%_ = 95% for the target volumes (defined as the total gross target volume GTV) and the minimization of the dose to all involved structures (including the remaining healthy brain and, according to the case, the eyeballs, the lenses, the optic nerves, the chiasm, the brainstem) according to the ALARA principle. For all patients 3 sets of plans were designed, optimized and computed, according to the following:

*HyperArc*: this is the technique under investigation. It was recently released for clinical use and its planning workflow consists of three phases:

i) Definition of the basic plan features: list of lesions and individual dose prescription (without limits in the number of possible targets, all with a possibly individual dose prescription);
ii) Selection of the arc geometry from a class solution of 5 non-coplanar arcs arranged with one single isocenter, automatically located according to the mutual distance between each lesion. Users can only select or de-select some arcs but cannot modify their trajectories (length and couch rotation).
iii) Automatic optimization of the collimator angle. This tool aims to orient the MLC leaves motion as much as possible along directions orthogonal to the ideal line connecting two or more lesions. This should result in the maximization of the dosimetric separation of the lesions and the reduction of the so-called dose bridging effect.
iv) The process can directly continue into the optimization and final dose calculation phases.

**Fig. 1:**
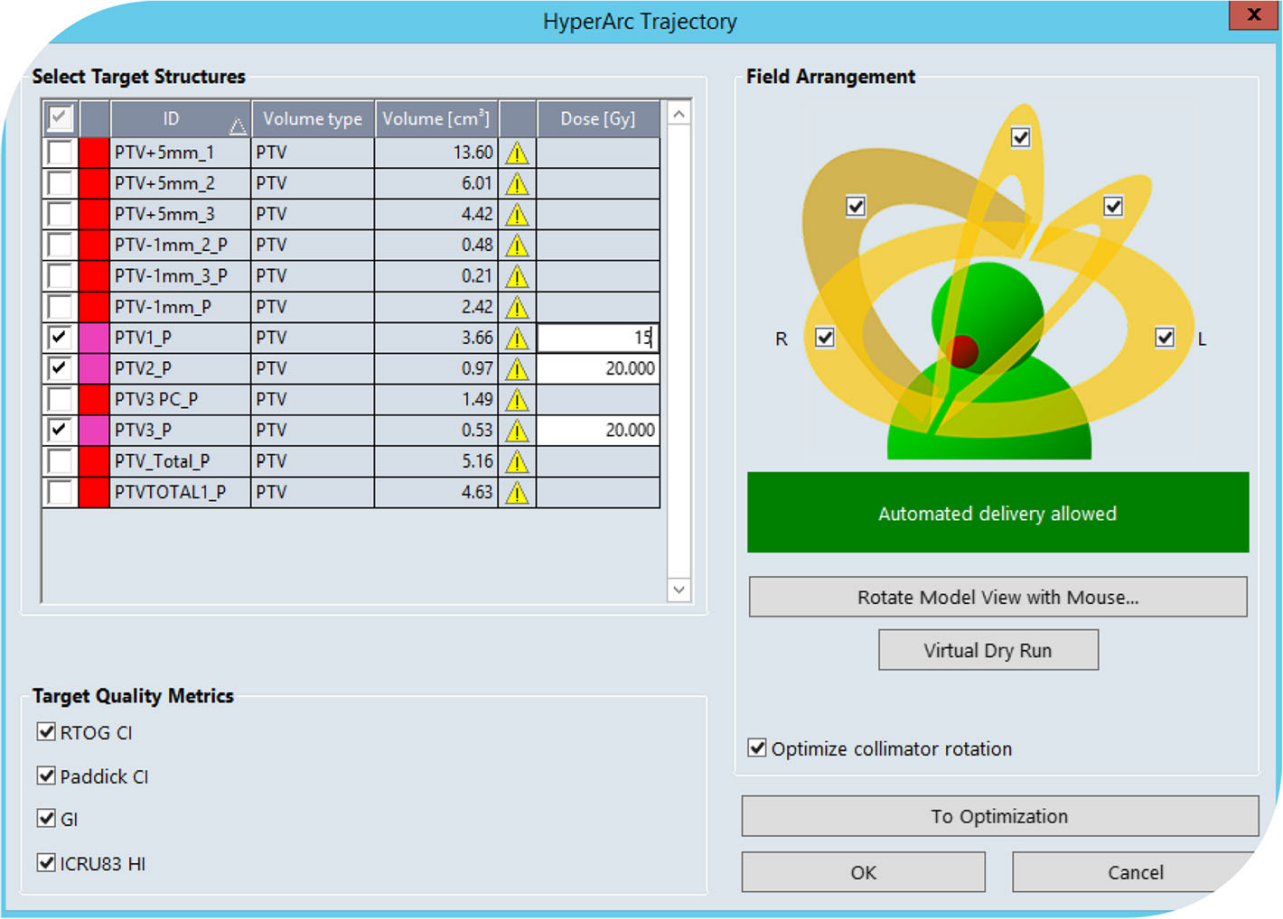
A screen shot of the user’s interface for the definition of the HyperArc treatment plan

Figure 1 shows a screen shot of the graphic interface used to generate the treatment plan arrangements. The arcs (up to five) are all connected into one automated sequence for the delivery, meaning that the entire treatment can be performed with one single virtual “beam-on” phase. During couch rotations no beam is active and the image guidance procedure requires that a full CBCT is acquired prior to the first arc with couch set at 0 degrees and planar 2D images can be further acquired prior of each arc. Optionally users can run an in silico dry run of the delivery to verify the absence of any risk of collision. Any change in the geometrical arrangements introduced by users, would void the HA nature of the plan and disable automation of delivery (as well as preventing optimization).

Plans were optimized in Eclipse (v.15.5) for the Edge delivery system equipped with a multileaf collimator with 2.5 mm resolution in the central region ((Varian Medical Systems, Palo Alto, USA). The Acuros dose calculation algorithm was used for the final calculation while the dedicated SRS version of the Photon Optimizer engine was applied for the inverse planning with a dose grid resolution of 1 mm. In the optimization two SRS specific tools were applied. Firstly the stereotactic normal tissue objective. This mimic what described in [13, 14] and automatically generates virtual shells around the targets to control and minimize the dose bridging effect between lesions and maximizes the steepness of the dose gradient. Secondly the automatic low dose objective (ALDO) option was selected to automate the management of the optimization priorities to the targets in order to achieve the same degree of coverage (ideally full coverage) to all lesions. The use of ALDO prevents the possibility to set upper constraints to the target volumes, differently from what doable with standard planning.

**Fig. 2:**
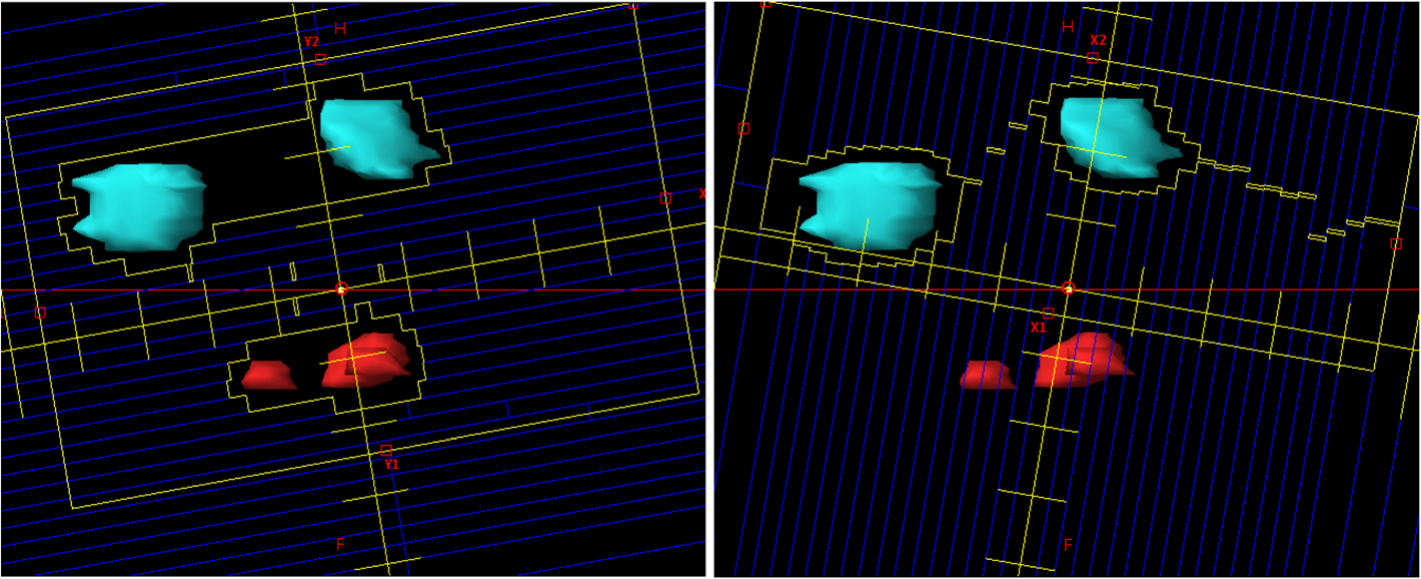
Conceptual visualization of the complexity of the manual arrangement of the collimator angle for the standard VMAT planning. On the contrary, the new HyperArc engine, includes an automatic optimization of the collimator angle

*VMAT*: Each VMAT plan, optimized in Eclipse with the RapidArc technique with the same treatment planning system as above (using the version 13.6), was optimized with a variable number of partial or full coplanar or non-coplanar arcs (from 2 to 5). A single isocenter strategy (to mimic as much as possible the HA approach) was applied to most of patients (8 out of 15). The remaining cases were planned with multiple isocentres in order to achieve plans which were at least considered clinically acceptable and not affected by severe sub-optimal dosimetric characteristics. The exact choice of the beam geometry was due by the number and location of the lesions with the general aim to minimize the involvement of the healthy structures in the brain. 6MV flattening filter free photon beams were chosen (with a maximum dose rate of 1400 MU/min) from a TrueBeam STX delivery platform (equipped with a MLC of 2.5 mm spatial resolution). For the final dose calculation, the same algorithm and resolution were applied as for the HA plans. To note that, for more than one lesions, the choice of the proper angle of collimation is not trivial. If tumors are placed in such way, that for any gantry angle there is no overlapping in the beam projection, the MLC directions should be set far less perpendicular to the arc plane. But for more sophisticated spatial placing the special set-up is required. As an example, Fig. 2 illustrates the case of a 5-tumors case and its problems of targets overlapping on BEV projection is shown below. There are two subsets of tumors: red ones and cyan ones, the red line is the projection of arc plane. The probe of MLC fitting for full arc to the all tumors failed. Left panel shows irradiation of large volume of health tissue placed between cyan tumors for a particular gantry angle. Any other collimator angle would cause similar problems.

In that case the better solution was to use two arcs, for cyan and red tumors independently (right panel, shown only MLC shape for cyan subset of targets). It seems that the best situation is when (1) the selected group of tumors rotates in one plane (some kind of tumors overlapping for projection occurs), (2) arc plane is perpendicular to the direction of leaf movements and (3) tumors are not concave in the linking direction for any projection. Sometimes a slight couch angle (less than 10 degrees) could give some improvement.

*Cyberknife*: Each CK plan was optimized with the Multiplan v.4.6 (Accuray Inc, USA) treatment planning system using the RayTracing algorithm, a dose calculation grid of 0.98 mm (the resolution of the CT dataset) was applied for the final calculations. The mean number of nodes per plan was 110 (ranging from 65 to 259) with 217 beam directions (152–351). For the collimation of the beams, both fixed size tubes or the Iris collimator were selected due to the different size, number and location of the lesions. The actual selection was performed according to the preference of the planners. For all plans, the dose cubes were exported in DICOM format and imported in the Eclipse system for planning comparison purposes.

### Quantitative and statistical analysis

Qualitative and quantitative analysis was performed on the dose distributions and the cumulative dose volume histograms (DVH) by computing a number of dose-volume metrics for the target volume and the organs at risk. For the targets, the analysis was carried out on the sum of all the targets, the near-to-minimum dose was defined as D_98%_ and the homogeneity as: (D_2%_-D_98%_)/D_prescription_. For the organs at risk the mean or the near-to-maximum doses were accounted for. The Paddick conformity and gradient indexes were computed according to the original formulation [25, 26] for the total target volumes.

In addition to these parameters and in agreement with the methods described in [27], the Radiation Planning Index (RPI) was computed. This is defined as:

$$ RPI=\sqrt[k+n]{\prod \limits_{j=1}^k\left\{\prod \limits_{i=1}^n\left[\left(1-\frac{W_i\cdot \underset{0}{\overset{D_{i\max }}{\int }}{V}^{OaR_i} dD}{\underset{0}{\overset{D_{i\max }}{\int }}100 dD}\right)\cdot \frac{\underset{0}{\overset{D_{j\max }}{\int }}{V}^{PTV_j} dD}{\underset{0}{\overset{D_{j\max }}{\int }}100 dD}\cdot \left(1-\frac{SDev_j}{100}\cdot {W}_j\right)\right]\right\}} $$

Where: *k* is the number of PTV (or GTV) structures, *n* is the number of critical structures,$\underset{0}{\overset{D_{i\max }}{\int }}{V}^{OaR_i} dD$ is the area under the graph for the integral DVH for a given OaR_i_, D_imax_ is the maximum dose for the i-th structure of OAR among all compared plans, expressed in [Gy], *W*_*i*_ is the weight factor assigned to i-th structure OaR, $\underset{0}{\overset{D_{j\max }}{\int }}{V}^{PTV_j} dD$ is the area under the graph for the integral DVH for the PTV_j_, *D*_*jmax*_ is maximum dose for the j-th structure of PTV (or GTV) among all compared plans, expressed in [Gy], *SDev*_*j*_ is the standard deviation expressed as a percentage for the structure of PTV_j_ in the analyzed plan, *W*_*j*_ is the weight factor assigned to j-th - PTV. The weight describes the importance of the given structure. If OARs sparing is more important than PTV coverage then *W*_*i*_ > *W*_*j*_ (0 ≤ *W* ≤ 1).

When the critical structures receive 0% of the reference dose and the whole tumour volume is covered by 100% of the prescribed isodose and the dose distribution inside the target is homogeneous (*SDev* = 0) then RPI = 1. RPI = 0 when each OAR volume is covered with the homogeneous maximal dose or standard deviation *SDev* is equal to 1. In clinical practice RPI values are in the range of 0 to 1.

For each investigated parameter, significance of the differences observed among the techniques was computed with nonparametric Kruskal-Wallis tests and the threshold was set to 0.05.

## Results

**Fig. 3:**
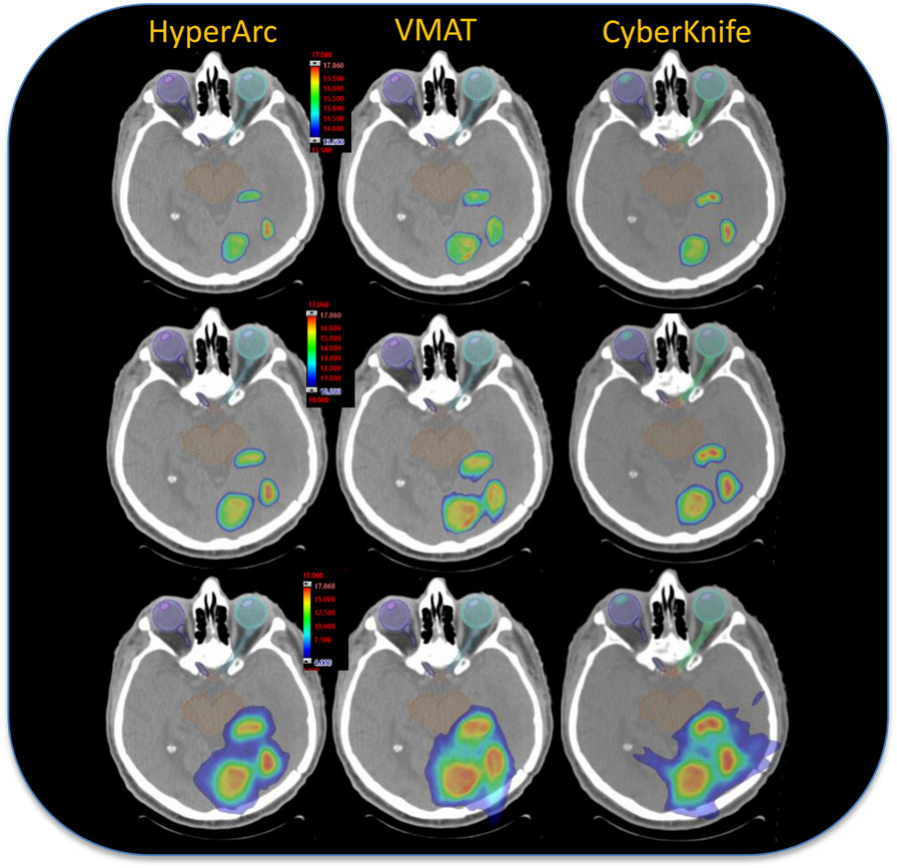
Axial views of the dose distribution for an example case with three lesions for the three different techniques, arranged in columns: HyperArc, VMAT, CyberKnife. The colorwash is set to 13.5 Gy (90% of the prescription) to 17.0 Gy – upper row, 10.0 Gy to 17.0 Gy – middle row, 4.0 Gy to 17.0 Gy – lower row. It is qualitatively noticeable the different management of the dose bridging and of the gradients between the different approaches

**Fig. 4:**
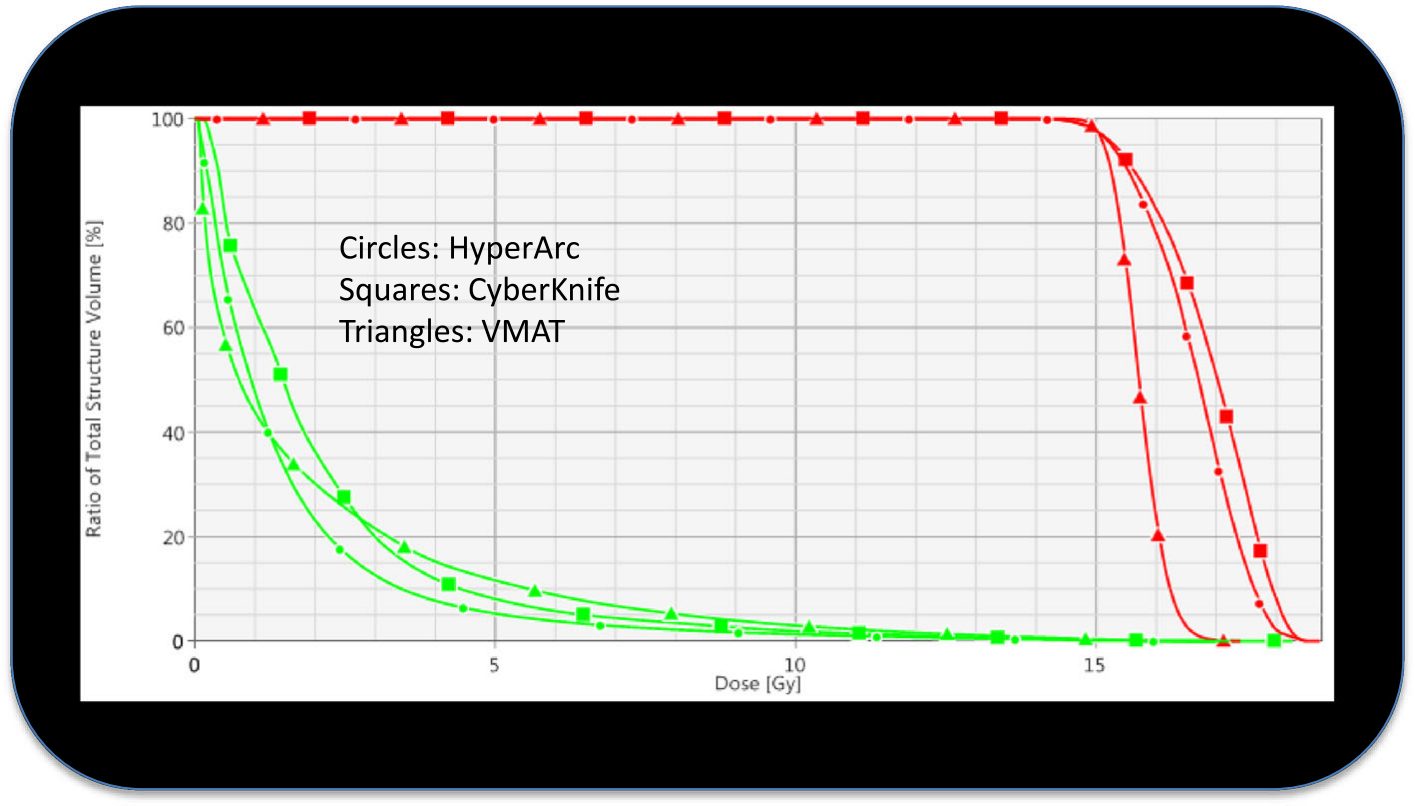
The cumulative dose volume histograms of the example case with three lesions for the three techniques under investigation. In red the total target volume, in green the healthy brain

Figure 3 presents the isodose distribution for a representative case (with 3 lesions) in three axial planes across the extension of the metastases for the three techniques under investigation. The dose prescription for this case was 15Gy and the color wash ranges from 13.5 (90% of the prescription) to 17.0 Gy - first row; from 10.0 Gy to 17.0 Gy – second row and from 4.0 Gy to 17.0 Gy in third row. Columns from left to right are HA, VMAT and CK respectively. From a qualitative perspective, the illustration demonstrates the differences in terms of gradients and of dose bridging mitigation among the various solutions. Figure 4 presents the cumulative DVH for the cumulative target volume and for the healthy brain for the same case. The data illustrates the reduction of the medium to high doses achieved by HA with respect to both techniques. Concerning the target, a compatible coverage was obtained while CK and HA presented an (equivalent) higher maximum dose which could be avoided in the VMAT case.

**Fig. 5:**
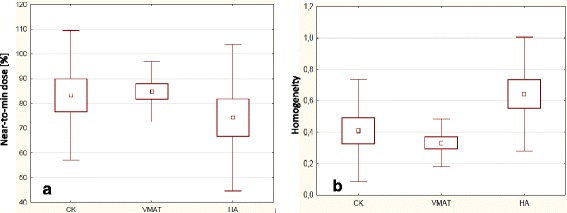
Summary of the analysis of the PTV data. **a** near-to-minimum dose; **b** homogeneity. The central point represents the mean, the solid box the standard error and the bars the standard deviation

Concerning the targets, Fig. 5a illustrates the boxplot relative to the near-to-minimum dose (in %) for all the techniques. The error bars correspond to 1 standard deviation, the solid box to the mean ± 1 standard error. No difference was found in this case (*p* = 0.28). Figure 5b provides the results for the homogeneity of the dose distributions. In this case a significant difference was observed between CK and HA (*p* = 0.02) and between VMAT and HA (*p* = 0.01). The Paddick conformity index resulted comparable for all techniques without statistically significant differences (it resulted 0.87 ± 0.07, 0.81 ± 0.09 and 0.86 ± 0.06 for CK, VMAT and HA respectively). No difference was observed among the techniques for the target volume receiving 90% of the planning dose while for the volume receiving 110% there was a significant difference (*p* = 0.03) between CK and HA or VMAT. The Paddick gradient index resulted comparable between CK and HA with no statistically significant difference although it was slightly better for HA while it was significantly worse for VMAT with respect to both the alternatives (5.5 ± 1.5, 6.3 ± 1.6 and 5.0 ± 1.4 respectively for CK, VMAT and HA).

**Fig. 6:**
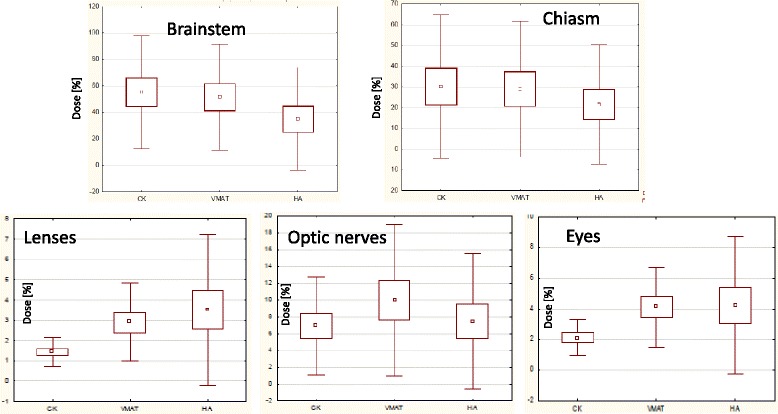
Summary of the analysis of the data for the organs at risk

Figure 6 summarizes the analysis on the organs at risk. No significant differences were observed for the brainstem, the chiasm and the optic nerves. Concerning the lenses, the difference between VMAT and HA was statistically significant (*p* = 0.02) while it resulted at the edge of significance between CK and HA (*p* = 0.05). Concerning the eyes although the mean findings suggest better results for CK, no significant difference was observed between CK and HA while this was the case when comparing CK vs. VMAT resulted in a *p* = 0.02.

**Fig. 7:**
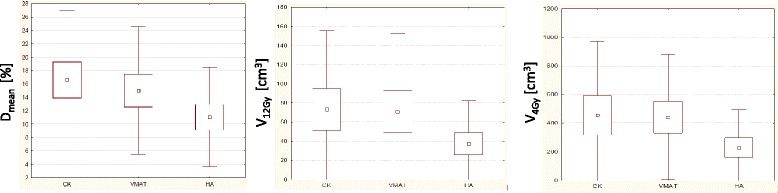
Summary of the analysis of the data for the healthy brain. In the first panel the mean dose is reported while in the second and in the third panels the V_12Gy_ and the V_4Gy_ data

Figure 7 illustrates the data for the healthy brain. In the first panel the mean dose is reported while in the second and in the third panel the V_12Gy_ and the V_4Gy_ data. As noticed, no difference was observed for the mean dose (*p* = 0.2) among the techniques. Only for V_4Gy_ differences are statistically significant (*p* = 0.01) for the median dose, the smallest median dose is for HA technique.

**Fig. 8:**
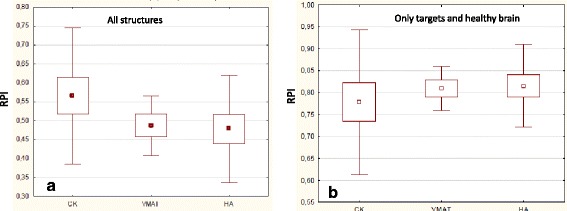
Summary of the analysis of the RPI. In the first panel when all the structures per each plan are included and in the second panel when only the targets and the healthy brain are considered

Figure 8 summarizes the analysis carried on by means of the RPI. In the first panel when all the structures per each plan are included and in the second panel when only the targets and the healthy brain are considered. In the first instance a near-to-significance difference was observed (*p* = 0.05) among the techniques while this was not the case in the second case (*p* = 0.62). To be noticed that a RPI value proximal to 1 means that the dose distributions meet ideally the planning objectives, while a RPI = 0 corresponds to the worst case. When all structures are included in the RPI calculation, CK resulted better (although at the edge of significance) while a substantial equicalence was observed when only the healthy brain was included as a relevant organ at risk.

The evaluation of the overall delivery time for the VMAT plans resulted in a range of 2.2 to 11.2 min (average: 5.9 ± 3.1 mins). In this case imaging and positioning time was not included since it might depend upon the protocols and the image guidance strategies. Since a single cone-beam CT acquisition requires about 3 min (acquisition and post-processing), its realistic to consider that an imaging plus verification time of 10–15 min might be added to the process.. Due to the automation of the delivery process and the integration of the imaging time, the total computed time required to complete a HA fraction resulted quite shorter than the corresponding time for VMAT. In fact, if the delivery time due to beam on is equivalent among the two approaches, the reduction of the dead times between arcs and the fast imaging process would enable the entire image guidance process to be completed in 5–10 min.

The total delivery time for CK plans resulted in a range from 37 to 76 min (with an average of 52.0 ± 10.4 mins). This calculated time is relative to a single fraction inclusive of kV imaging for positioning purposes. One CK plan was composed of six different plans and was not accounted above. For this summed plan the total time of all treatment sessions was 275 mins, assumed daily treatment of all the tumors.

## Discussion

A planning study aiming to compare three different techniques for the radiosurgical treatment of multiple brain metastases was carried out. CK, VMAT and the newly clinically introduced HA (an evolution of the VMAT from the planning and delivery point of view) were compared on a cohort of 15 patients. No experimental verification of the dose distributions was performed and included in the study since beyond the purpose to the project. Nevertheless, both CK and VMAT are consolidated techniques (and in this perspective HA falls in the category of the VMAT delivery) and this point has been already proven by the clinical practice. However, it is important to keep in mind the different therapeutic dose definition in VMAT and CK technique [28].

Concerning the field of the in-silico comparison, the dose distributions optimized for the three techniques under investigation, namely CK, VMAT and HA techniques resulted comparable from a clinical perspective. The main remarkable difference was observed for the estimated treatment time, significantly higher for CK compared to the VMAT based approaches. The delivery time for HA is further reduced compared to the VMAT. The mean exposition time (when beam is on) is for CK - about 35 mins, for VMAT - 3 mins and 4 mins for HA technique.

Concerning the analysis of the dose distributions, the best homogeneity was observed for the VMAT and the worst with HA with CK falling nearer to VMAT; the observed differences were statistically significant. Regarding the organs at risk, the main discrepancies among techniques were observed for the lensed and the eyes while no remarkable dependency was observed for the brainstem, the chiams and the optic nerves. In the case of the lenses and the eyes, the average findings demonstrate better sparing for CK. The absence or limed value of the statistical significance of the observed discrepancy might depend also on the large inter-patient variability observed in HA or VMAT (large standard deviations), much less marked for CK.

HA allowed also to better spare the healthy brain (defined as the brain minus the targets) in the very low dose region (V_4Gy_) while no differences were observed for V_12Gy_ and RPI. Volumes which receive 4 and 12 Gy (V_4Gy_ and V_12Gy_) are the smallest for HA technique. In each plan, dose 4 and 12 Gy have the smallest volume for HA technique, but only for V_4Gy_ differences are statistically significant for the median dose. It is important to remember that 4 (or 10) Gy given in one fraction would result in different biological effect than given in two or more fractions.

The multiparametric nature of the OAR sparing (and target goverage) makes hard to identify a single reason to justify the difference in sparing between lenses and eyes compared to the healthy brain. Certainly, the technical implementation of HA inherently contributes to a better sparing of the tissue surrounding the targets, and this is mostly given by the healthy brain in the patients we investigated; but this not necessarily at the expense, as a trade-off, of reduced sparing capability of the other structures. On the other hand, the quasi-blind planning methodology adopted for the comparison, could play a role in the different sparing of the eyes and the lenses. In all cases and for all techniques, the dosimetric findings were within planning aims which only required to minimize the dose to the OARs without the imposition of numerical fixed objectives. For example, the average near to maximum dose to the lenses (Fig. 6) resulted “low” for all techniques, below the normally clinically accepted threshold of 10Gy. Different efforts in the optimization process might have better harmonized the differences between the techniques. This might be considered as a limitation of the study design but it reflects the clinical practice in the home institute.

With different fractionation schemes it might have been possible to report either biologically equivalent doses (i.e. corrected per fractionation) of to report relative doses. Both approaches would lead to somehow similar conclusions although with numerical differences. Nevertheless, the calculation of biological equivalent doses in the stereotactic frame would be affected by some additional uncertainty due to the calculation parameters, while the relative report would have partially masked the possibility to directly correlate the results from different techniques vs possible clinical implications. We opted to report the results in terms of the volume of healthy brain receiving absolute levels (4 or 12Gy) of physical dose being this an objective quantity, irrespectively from fractionation. Of course, the clinical relevance of the findings should have to be proven in a clinical experiment.

The analysis of the RPI metric indicated that the plans made for the CK technique are closest to the planning expectations if all the structures are taken into account but at the boundary of statistical significance (*p* = 0.05). If only the targets and the healthy brain are considered, then all the three techniques resulted basically equivalent. These results support two arguments. Firstly, a tool like RPI would require the availability of plans optimized with very stringent criteria for all the included structures (and this was not sufficient for some of the organs at risk as discussed above). Secondly, limiting to the two primary structures (target and healthy brain), all three techniques resulted very comparable in relative quality. It is also important to mention that, quality based metrics like the RPI, are subject to some arbitrariety. In fact, if in the RPI formula the weighting factor for the healthy brain would be increased, giving more relevance to this structure, then it would be possible to “over” express the relevance of one particular structure and bias the global metric.

As a conclusive remark, it is important to stress that, as in all planning comparisons, it is difficult to manage different systems (and optimization engines) in a completely coherent way. This would be necessary ideally in order to guarantee that all the parameters are ta least maintained except the ones which can be improved or which are fundamental for the study. As mentioned, differences in the algorithms and in the interfaces, as well as in the planners preferences, cannot be completely mitigated or eliminated. The current study was designed in a way to possibly minimize all these effects but some could still be present as discussed but should not invalidate the global message of substantial equivalence among the different approaches.

## Conclusions

The recently released HA method for the SRS irradiation of multiple metastases was investigated in comparison to CK and VMAT techniques. From a dosimetric standpoint, all techniques proved to be comparable from a clinical viewpoint while the integrated and automated HA suggested a remarkable improvement in the delivery time. A comprehensive quality metrics for the plans, restricted to the analysis of the healthy brain and the target volumes, proved that HA can maximize the sparing of the brain without compromising the target coverage. Further studies and clinical reports are required to confirm these early observations.

## Abbreviations

ALARA: As low as reasonably achievable
CK: CyberKnife
CT: Computed tomography
CTV: Clinical target volume
HA: HyperArc
PTV: Planning target volume
RA: RapidArc
RPI: Radiation planning index
SRS: Stereotactic radiosurgery
VMAT: Volumetric modulated arc therapy

## References

[1] Halasz L, Uno H, Hughes M, D’Amico T, Dexter E, Edge S, Hayman J, Niland J, Otterson G, Pisters K, Theriolt R, Weeks J, Punglia R (2016). Comparative effectiveness of stereotactic radiosurgery versus whole brain radiation therapy for patients with brain metastases from breast or non small cell lung cancer. Cancer.

[2] Jairam V, Chiang V, Yu J, Knksely J (2013). Role of stereotactic radiosurgery in patients with more than four brain metastases. CNS oncol.

[3] Elaimy A, Mackay A, Lamoreaux W, Fairbanks R, Demakas J, Cook B, Lee C (2011). Clinical outcomes of stereotactic radiosurgery in the treatment of patients with metastatic brain tumors. World Neurosurg.

[4] Soliman H, Das S, Larson D, Sahgal A. Stereotactic radiosurgery (SRS) in the modern management of patients with brain metastases. Oncotarget. 2016;7:12318–30. 10.18632/oncotarget.7131 PMC4914287 26848525

[5] Lam T, Sahgal A, Cahng E, Lo S (2014). Stereotactic radiosurgery for multiple brain metastases. Exp Rev Anticancer Ther.

[6] Dong P, Lee P, Ruan D, Long T, Romeijn E, Yang Y (2013). 4π non-coplanar liver SBRT: a novel delivery technique. Int J Radiat Oncol Biol Phys.

[7] Dong P, Lee P, Ruan D, Long T, Romeijn E, Low D (2013). 4π non-coplanar stereotactic body radiation therapy for centrally located or larger lung tumors. Int J Radiat Oncol Biol Phys.

[8] Nguyen D, Rwigema J, Yu V, Kaprealian T, Kupelian P, Selch M (2014). Feasibility of extreme dose escalation for glioblastoma multiforme using 4π radiotherapy. Radiat Oncol.

[9] Dong P, Nguyen D, Ruan D, King C, Long T, Romeijn E (2014). Feasibility of prostate robotic radiation therapy on conventional c-arm linacs. Pract Radiat Oncol.

[10] Woods K, Nguyen D, Tran A, Yu V, Cao M, Niu T (2016). Viability of non-coplanar VMAT for liver SBRT compared with coplanar VMAT and beam orientation optimized 4π IMRT. Adv Radiat Oncol.

[11] Yu V, Tran A, Nguyen D, Cao M, Ruan D, Low D, Sheng K (2015). The development and verification of a highly accurate collision prediction model for automated non-coplanar plan delivery. Med Phys.

[12] Rwigema J, Nguyen D, Heron D, Chen A, Lee P, Wang P (2015). 4p non-coplanar stereotatic body radiation therapy for head and neck cancer: potential to improve tumor control and late toxicity. Int J Radiat Oncol Biol Phys.

[13] Clark G, Popple R, Prendergast B, Spencer S, Thomas E, Stewart J, Guthrie B, Markert J, Fiveash J (2012). Plan quality and treatment planning techniques for single isocenter cranial radiosurgery with volumetric modulated arc therapy. Prat Radiat Oncol.

[14] Thomas E, Popple R, Wu X, Clark G, Markert J, Guthrie B, Yuan Y, Dobelbower M, Spencer S, Fiveash J (2014). Comparison of plan quality and delivery time between volumetric arc therapy (RapidArc) and Gamma Kinife radiosurgery for multiple cranial metastases. Neurosurgery.

[15] Mayo CS, Ding L, Addesa A, Kadish S, Fitzgerald TJ, Moser R (2010). Initial experience with volumetric IMRT (RapidArc) for intracranial stereotactic radiosurgery. Int J Radiat Oncol Biol Phys.

[16] Macdougall ND, Dean C, Muirhead R (2014). Stereotactic body radiotherapy in prostate cancer: is rapidarc a better solution than cyberknife?. Clin Oncol (R Coll Radiol).

[17] Lin YW, Lin KH, Ho HW, Lin HM, Lin LC, Lee SP, Chui CS (2014). Treatment plan comparison between stereotactic body radiation therapy techniques for prostate cancer: non-isocentric CyberKnife versus isocentric RapidArc. Phys Med.

[18] Lin YW, Lin LC, Lin KL (2014). The early result of whole pelvic radiotherapy and stereotactic body radiotherapy boost for high-risk localized prostate cancer. Front Oncol.

[19] Paik EK, Kim MS, Choi CW, Jang WI, Lee SH, Choi SH, Kim KB, Lee DH (2015). Dosimetric comparison of volumetric modulated arc therapy with robotic stereotactic radiation therapy in hepatocellular carcinoma. Radiat Oncol J.

[20] Yang J, Ma L, Wang XS, Xu WX, Cong XH, Xu SP, Ju ZJ, Du L, Cai BN, Yang J (2016). Dosimetric evaluation of 4 different treatment modalities for curative-intent stereotactic body radiation therapy for isolated thoracic spinal metastases. Med Dosim.

[21] Seppälä J, Suilamo S, Tenhunen M, Sailas L, Virsunen H, Kaleva E, Keyriläinen J (2017). Dosimetric comparison and evaluation of 4 stereotactic body radiotherapy techniques for the treatment of prostate Cancer. Technol Cancer Res Treat.

[22] Nalichowski A, Kaufman I, Gallo J, Bossenberger T, Solberg T, Ramirez E, Yan Y, Fredrick J, Bichay T, Mayville A, Burmeister J (2017). Single fraction radiosurgery/stereotactic body radiation therapy (SBRT) for spine metastasis: a dosimetric comparison of multiple delivery platforms. J Appl Clin Med Phys.

[23] Bae SH, Kim MS, Jang WI, Kim KB, Cho KH, Kim WC, Lee CY, Kim ES, Choi CW, Chang AR, Jo S, Kim JY (2017). Quality assurance for a multicenter phase II study of stereotactic ablative radiotherapy for hepatocellular carcinoma ≤5 cm: a planning dummy run. Jpn J Clin Oncol.

[24] Liu H, Andrews D, Evans J, Werner-Wasik M, Yu Y, Dicker A, Shi W (2016). Plan quality and treatment efficiency for radiosurgery to multiple brain metastases: non coplanar RapidArc vs. Gamma knife. Front Oncol.

[25] Paddick I (2000). A simple scoring ratio to index the conformity of radiosurgical treatment plans. J Neurosurg.

[26] Paddick I, Lippitz B (2006). A simple dose gradient measurement tool to complement the conformity index. J Neurosurg.

[27] Slosarek K, Grzadziel A, Szlag M, Bystrzycka J (2008). Radiation planning index for dose distribution evaluation in stereotactic radiotherapy. Rep Pract Oncol Radiother.

[28] Ślosarek K, Kopczyńska J, Osewski W (2016). Dose specification in external beam radiotherapy for CyberKnife and VMAT techniques applied to a case of prostate cancer. Nowotwory J Oncol.

